# Supplementary material for: Effect and Mechanism of Pitch Coating on the Rate Performance Improvement of Lithium-Ion Batteries
Source: Materials (Basel). 2022 Jul 5;15(13):4713. doi: 10.3390/ma15134713 (PMC9268190; doi:10.3390/ma15134713)
Supplement: Supplementary file 1 [file materials-15-04713-s001.zip › materials-1703850-supplementary.pdf]

Table S1. The content of the pitch calculated using the density difference.

	True density (g/ml)	*Pitch contents (%)
FG	2.310	0
CFG5	2.289	4.10
CFG10	2.274	7.03
CFG15	2.265	8.92
CFG20	2.245	13.00
Pitch (900 °C)	1.810	100

* [density of FG - density of samples]/[density of FG - density of pitch (900 °C)]

Table. S2. Elemental analysis of coating pitch.

	Carbon	Hydrogen	Nitrogen	Sulfur
Raw	93.39	5.45	0.012	0.090
900 °C	96.48	0.69	0.077	0.059

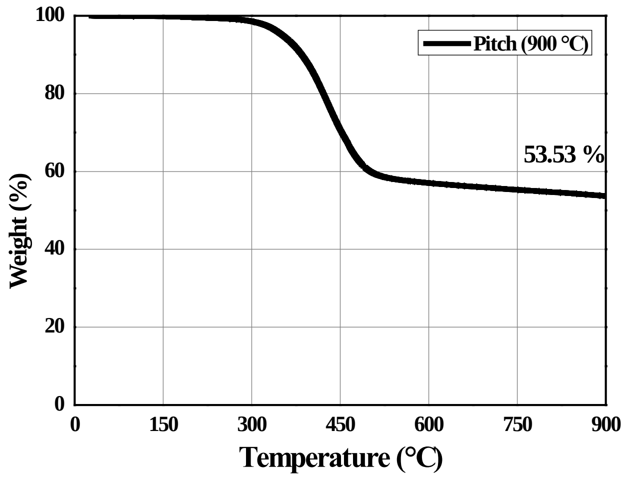

Figure S1. Thermal behavior of coating pitch in N_2 atmosphere.

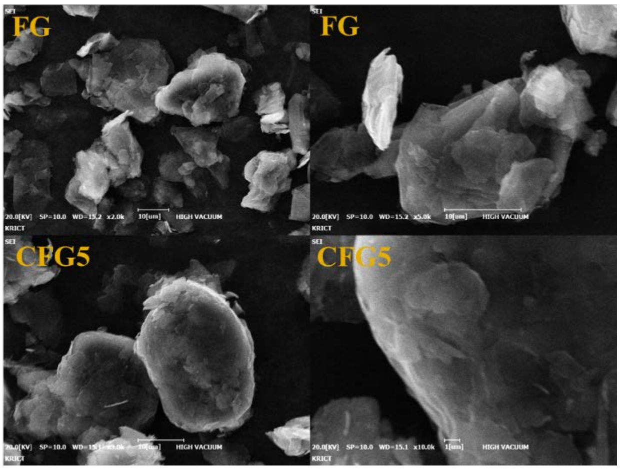

Figure S2. SEM images of FG and CFG5.

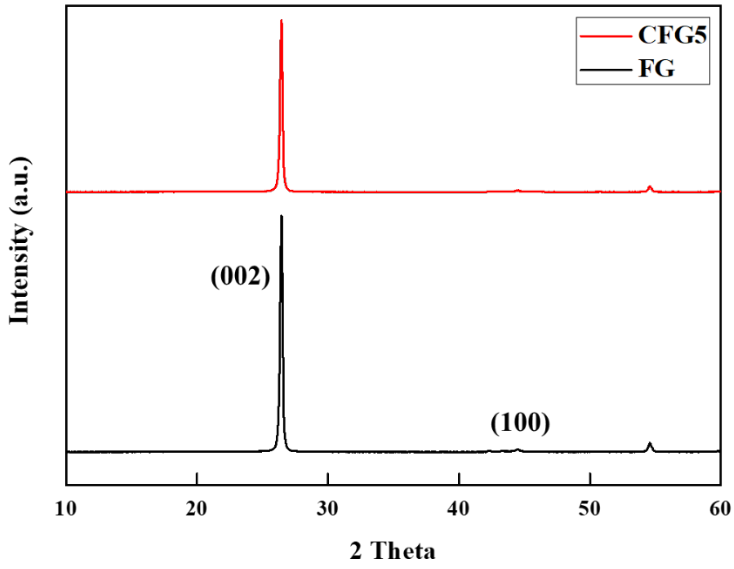

Figure S3. XRD of FG and CFG5.

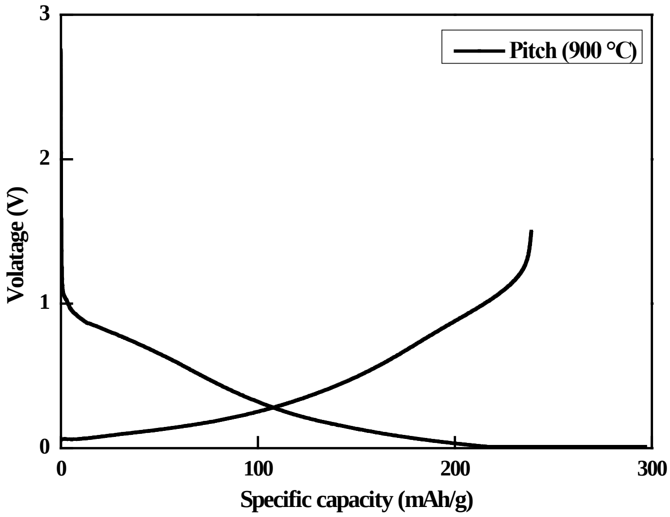

Figure S4. Galvanostatic charge/discharge (GDC) of pyrolyzed pitch at the first cycle.

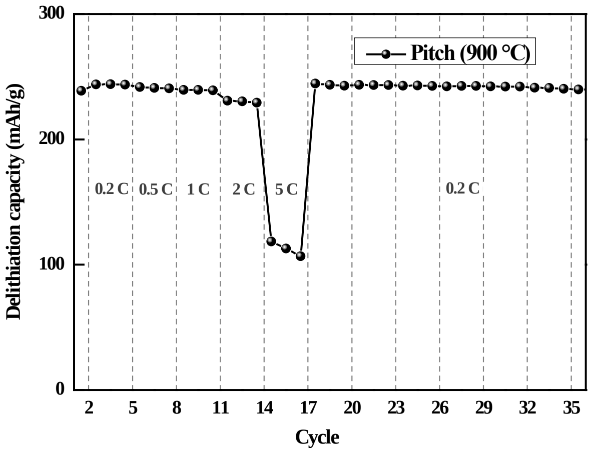

Figure S5. Rate performance at various delithiation rates of pyrolyzed pitch and cycling stability.

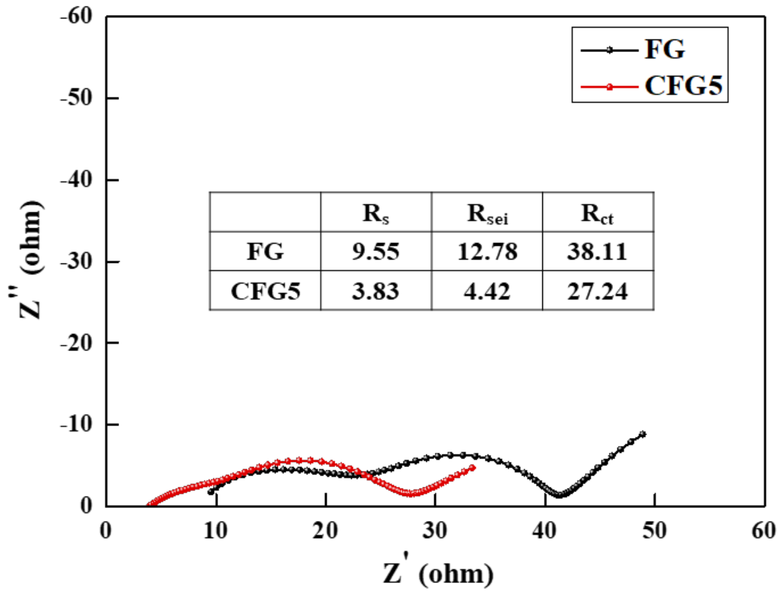

	R_s	R_{sei}	R_{ct}
FG	9.55	12.78	38.11
CFG5	3.83	4.42	27.24

Figure S6. EIS curves after cycling of FG and CFG5.

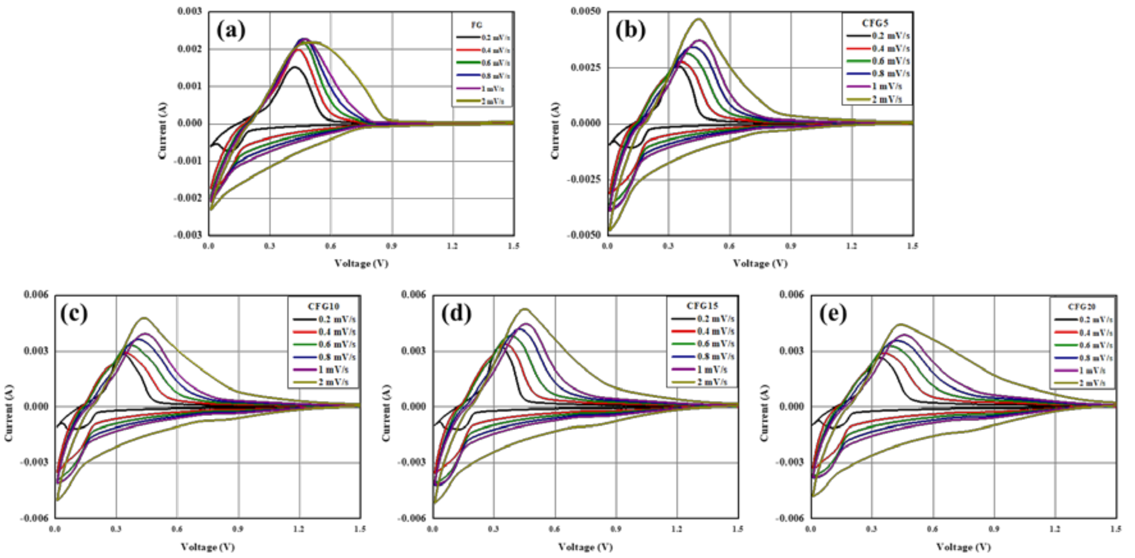

Figure S7. Cyclic voltammograms oat different scan rates of (a) FG, (b) CFG5, (c) CFG10, (d) CFG15, and CFG20.

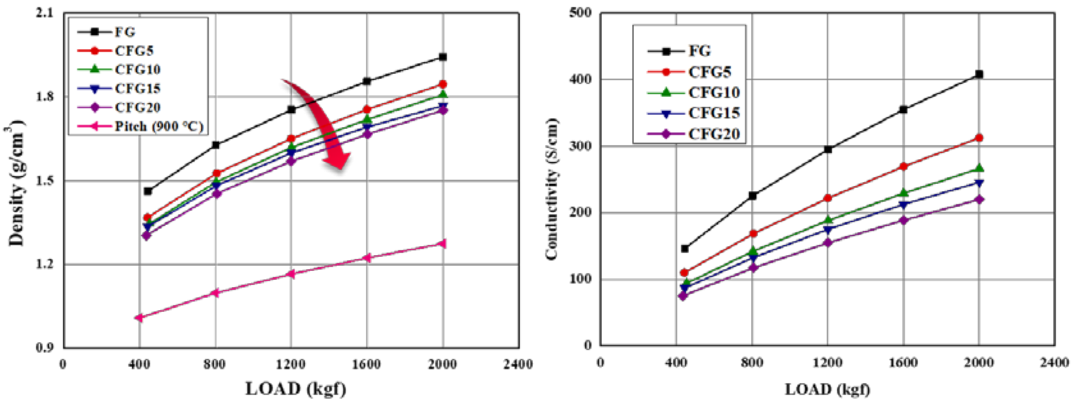

Figure S8. Influence of (a) density and (b) electronic conductivity as a load (kgf).

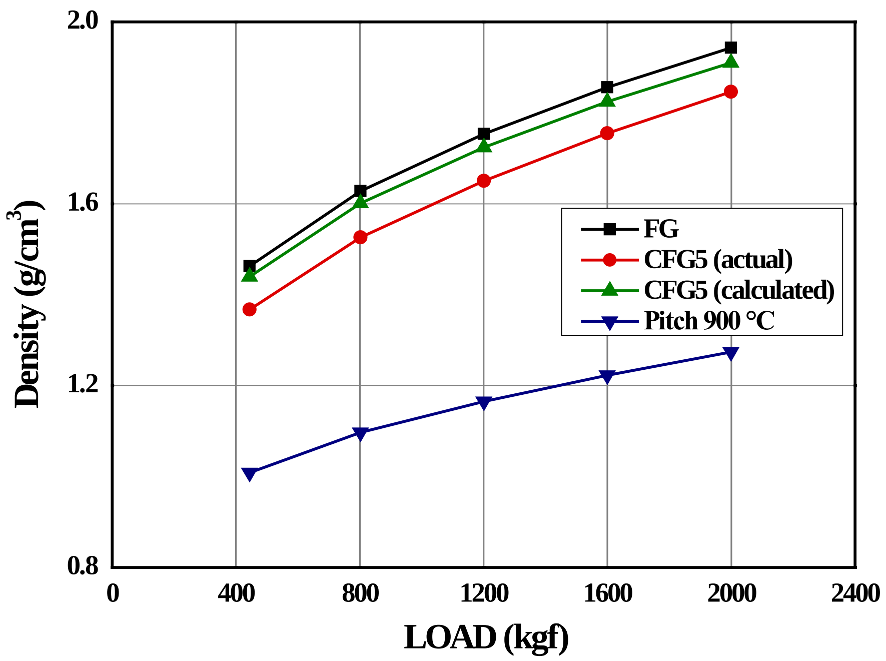

Figure S9. Actual density and the calculated density of CFG5 and actual density of FG and pyrolyzed pitch according to the load.

Table. S3. Actual density and the calculated density according to the load.

	Packing density (g/cm^3)							
	CFG5		CFG10		CFG15		CFG20	
LOAD (kgf)	Actual value[a]	Calculated value[b]	Actual value	Calculated value	Actual value	Calculated value	Actual value	Calculated value
400	1.367	1.440	1.346	1.417	1.335	1.394	1.304	1.372
800	1.526	1.601	1.495	1.575	1.482	1.548	1.453	1.522
1200	1.651	1.724	1.619	1.695	1.599	1.666	1.569	1.636
1600	1.755	1.824	1.719	1.793	1.692	1.761	1.666	1.729
2000	1.846	1.910	1.808	1.876	1.769	1.843	1.752	1.809

[a]Actual value was measured using a powder resistivity measurement system.

[b]Calculated value was estimated by the equation (density of FG at Load) x (weight percent of FG) + (density of Pitch at Load) x (weight percent of pitch).
